# Impact of Vacuum-Assisted Closure (VAC) Therapy on Clinical Outcomes of Patients with Sternal Wound Infections: A Meta-Analysis of Non-Randomized Studies

**DOI:** 10.1371/journal.pone.0064741

**Published:** 2013-05-31

**Authors:** Matthew E. Falagas, Giannoula S. Tansarli, Anastasios Kapaskelis, Konstantinos Z. Vardakas

**Affiliations:** 1 Alfa Institute of Biomedical Sciences (AIBS), Athens, Greece; 2 Department of Internal Medicine - Infectious Diseases, Mitera Hospital, Hygeia Group, Athens, Greece; 3 Department of Medicine, Tufts University School of Medicine, Boston, Massachusetts, United States of America; Università Vita-Salute San Raffaele, Italy

## Abstract

**Objective:**

To examine the impact of VAC therapy on mortality of patients with sternal wound infections after cardiothoracic surgery.

**Summary Background Data:**

Controversial results regarding mortality of patients with sternal wound infections were published.

**Methods:**

We performed a systematic search in PubMed and Scopus. Mortality was the primary outcome of the meta-analysis. Recurrences, complications and length of stay were secondary outcomes.

**Results:**

Twenty-two retrospective studies including 2467 patients were eligible for inclusion. Patients treated with VAC had significantly lower mortality compared to those treated without VAC [2233 patients, RR = 0.40, (95% CI 0.28, 0.57)]. This finding was consistent regardless of the study design, the exclusion of studies with positive findings, the criteria for establishment of the compared groups, the time of mortality assessment or the type of infections under study, provided that adequate data was available. VAC therapy was associated with fewer recurrences (RR = 0.34, 95% CI: 0.19–0.59). The meta-analysis did not show any difference in the length of stay (RR = −2.25, 95% CI: −7.52–3.02).

**Conclusions:**

VAC therapy was associated with lower mortality than other surgical techniques in retrospective cohorts of patients with DSWIs following cardiothoracic surgery.

## Introduction

Deep sternal wound infections (DSWI), namely mediastinitis and osteomyelitis, are a serious complication occurring in 1% to 5% of patients after cardiothoracic operations in individual studies.[1], [2] Intravenous antibiotics and several surgical techniques had been used in the past for their treatment; however, they have been associated with increased short- and long-term mortality.[3] A new technique using topical negative pressure by controlled suction has been introduced in the treatment of wounds achieving wound closure through the formation of granulation tissue. This technique, most commonly applied by vacuum-assisted closure (VAC) wound therapy system, has gradually gained ground and replaced most of the conventional types of wound treatment due to the faster wound healing,[4], [5], [6] lower length of hospital stay[7], [8], [9] and the subsequent lower in-hospital cost.[6], [10].

Moreover, early studies showed that VAC therapy has the potential to reduce both in-hospital and long-term mortality.[11], [12] A meta-analysis published in 2011 showed that patients treated with VAC had shorter duration of hospitalization but no difference in mortality compared to those treated with a non-VAC therapy.[13] Since this publication several new studies became available that expanded our knowledge regarding the effectiveness of VAC application for the treatment of sternal wound infections. We aimed to systematically review and synthesize the available evidence with the methodology of meta-analysis in order to examine the impact of VAC therapy on mortality of patients with sternal wound infections.

## Methods

### Literature Search

We performed a systematic search in PubMed and Scopus electronic databases in September 2012. The search term that was applied in PubMed was the following: (“negative pressure” OR vac OR “vacuum assisted”) AND (wound) AND (infection). A more conservative search term was applied in Scopus database: ("negative pressure" OR vac OR "vacuum assisted") AND (sternal wound infection OR dswi OR mediastinitis OR osteomyelitis). In addition, the bibliographies of all relevant articles were searched in order to identify further potentially eligible studies. Articles written in a language other than English, German, French, Spanish, Italian or Greek were not evaluated. Only published studies were included; abstracts from conferences were excluded.

### Study Selection

Articles reporting the comparative outcomes of patients with sternal wound infections treated with VAC versus a non-VAC therapy were considered eligible for the meta-analysis regardless of the study design, patient characteristics, type of surgery and additional used interventions, deep or superficial sternal wound infections. When a patient population was included in more than one published studies, only the study with the bigger total study population was included. Studies focusing primarily in sternal wounds without infections or other types of wounds were excluded.

### Data Extraction

Data was extracted regarding the major characteristics of the included studies (first author, country, period of the study, study design), number of patients in each treatment arm, group establishment regarding the type of therapy selected, patient co-morbidity regarding the cardiothoracic operations conducted, and time of mortality assessment.

### Definitions and Outcomes

Sternal wound infections could comprise both deep and superficial infections developed after a cardiothoracic surgery. The definition of osteomyelitis, mediastinitis and superficial wound infections was based on the definitions provided by the selected individual studies.

The primary outcome of the review was in-hospital mortality. When in-hospital mortality was not provided by the authors of a study, 30-day or 90-day mortality was selected. Secondary outcomes comprised recurrence, as defined by the authors of the included studies, and hospital length of stay (LOS).

### Statistical Analysis

The non-randomized studies that were analyzed were considered to be heterogeneous by definition and therefore, the Mantel-Haenszel random effects model (REM) was applied. Pooled risk ratios (RR) and 95% confidence intervals (CI) were calculated regarding all outcomes. Statistical heterogeneity between studies was assessed by using the *χ* ^2^ test (p<0.10 was defined to indicate the presence of heterogeneity) and the *I* ^2^ (for assessing the degree of heterogeneity). The meta-analysis was performed with Review Manager for Windows, version 5.1.

## Results

**Figure 1:**
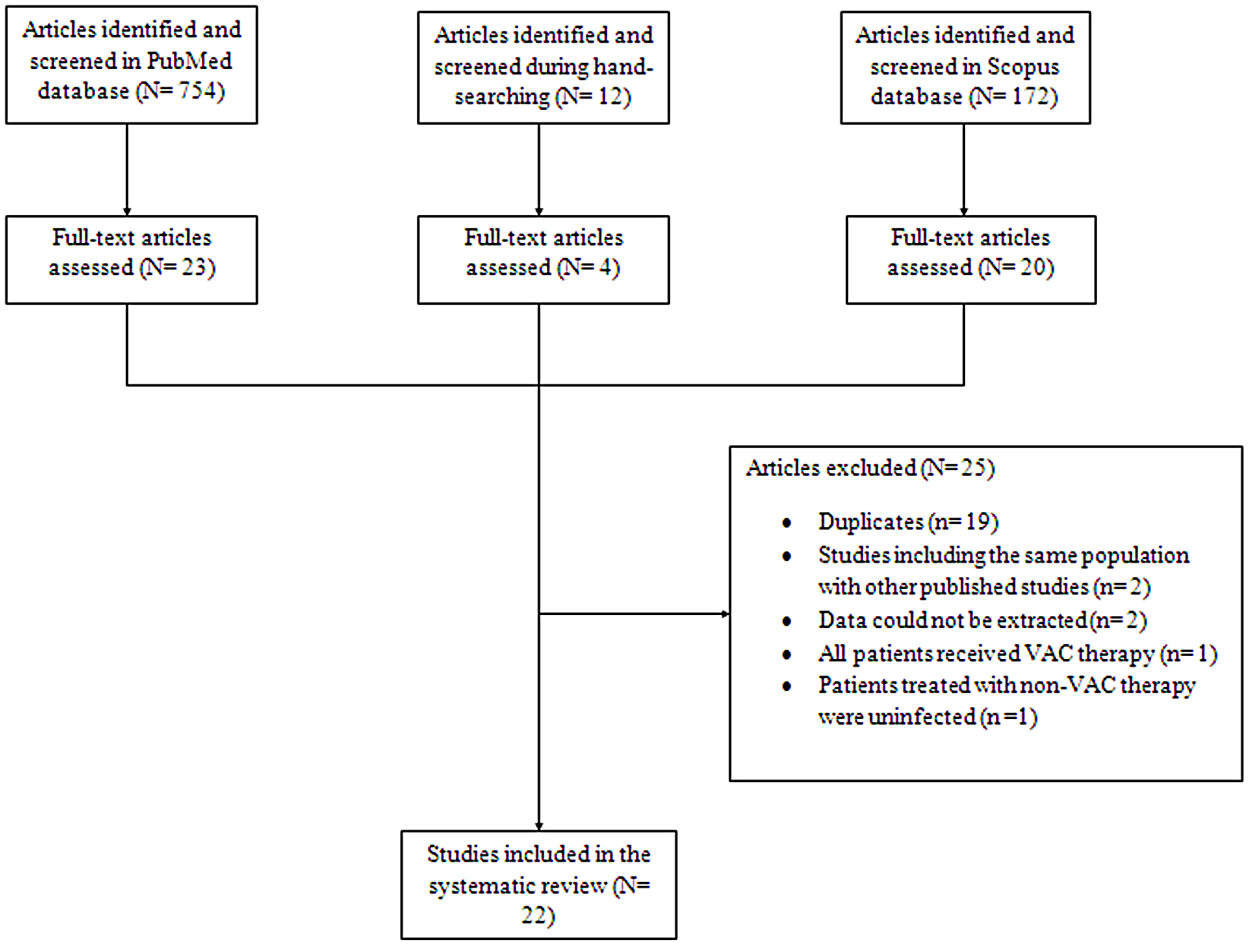
Flow diagram of the systematic search and study selection process.

The systematic search in both databases generated 938 articles (754 PubMed, 172 Scopus, 12 hand-searching). The selection process that was followed for the inclusion of the studies is depicted in Figure 1. Twenty-two studies were finally included in the review.[8], [11], [14], [15], [16], [17], [18], [19], [20], [21], [22], [23], [24], [25], [26], [27], [28], [29], [30], [31], [32], [33] The characteristics of the included studies are presented in Table 1. Twenty one studies reported on deep sternal wound infections (16 on mediastinitis,[11], [14], [15], [16], [17], [18], [19], [20], [21], [23], [26], [27], [28], [30], [32], [33] two on osteomyelitis[8], [25] and 3 did not specify the type of infections[24], [29], [31]). One study included both deep (69%) and superficial (31%) sternal wound infections.[22] All included studies were retrospective cohorts. Thirteen of the 22 studies provided data with regard to the VAC system that was applied and in all of them the KCI (Kinetic Concepts, Inc.) system was used.[8], [14], [17], [18], [19], [21], [22], [23], [24], [28], [30], [31], [32] Data regarding funding was provided by 4 studies;[11], [18], [22], [32] only one was industry funded.[22].

**Table 1:** Characteristics of the studies included in the meta-analysis**.**

First authorYear	Study design; period, country	Number of analyzed patients (VAC vs non-VAC)	Patient co-morbidity	Group establishment	System of VAC used	Mortality assessed at	Funded or non-funded study
Deniz2012^19^	Retrospective cohort; 2000–2011, Turkey	90 (47 vs 43)	60% coronary artery bypass revascularization, isolated 32% valvular procedure, 8% valvular in combination with coronary bypass procedures	2000–2003: non-VAC2003–2011: VAC	KCI system	90-day	NR
Fleck2012^22^	Retrospective cohort; 1995–2011, Austria	524 (326 vs 198)	Cardiac operations (VAC group: 62% aorto-coronary artery bypass, 39% VR, 17% congenital surgery or aortic surgery or heart transplantation)	1995–2001: non-VAC2002–2011: VAC	KCI system	Undetermined	KCI, USA
Risnes2012^28^	Retrospective cohort; 1997–2010, Norway	104 (64 vs 66)	CABG	1997–2002: non-VAC2002–2006: both non-VAC and VAC2006–2010: VAC	KCI system	30-day	NR
Rodriguez Cetina Biefer2012^29^	Retrospective cohort; 1999–2008,Portugal	159 (105 vs 54)	51% CABG, 18% isolated valve, 18% CABG/valve, 14% other procedures (thoracic aneurysms, aortic dissections, congenital repair procedures)	According to the surgeon’s discretion; VAC available for use at the clinic after 2002	NR	NA	NR
Simek2012^31^	Retrospective cohort; 2002–2007, Czech Republic	62 (34 vs 28)	76% CABG, 6% valve, 18% CABG+valve	2002–2004: non-VAC2004–2007: VAC	KCI system	In-hospital	NR
Steingrimsson2012^32^	Retrospective cohort; 2000–2010, Iceland	43 (20 vs 23)	63% CABG, 16% AVR+CABG, 7% aortic valve replacement alone	2000–2005: non-VAC2005–2010: VAC	KCI system	In-hospital	Landspitali University Research Foundation
Vos2012^33^	Retrospective cohort; 2000–2011, Netherlands	132 (89 vs 43)	81% CABG, 75% LIMA, 21% RIMA, 22% AVR, 8% MVR	VAC or non-VAC; no reasons are reported	NR	In-hospital	NR
Assmann2011^14^	Retrospective cohort; 2004–2008, Germany	154 (82 vs 72)	68% CABG	VAC or non-VAC; no reasons are reported	KCI system	In-hospital	NR
De Feo2011^18^	Retrospective cohort; 1979–2009, Italy	200 (55 vs 145)	57% coronary artery bypass, 26% valve surgery, 18% other procedures	1979–2002: non-VAC2002–2009: VAC	KCI system	In-hospital	Non-funded
Kobayashi2011^25^	Retrospective cohort; 2001–2007, Japan	16 (9 vs 7)	44% CABG, 19% AVR, 31% thoracic aortic surgery, 6% cardiac trauma	2001–2003: non-VAC2003–2007: VAC	NR	Undetermined	NR
Morisaki2011^26^	Retrospective cohort; 1991–2010, Japan	59 (8 vs 51)	58% CABG, 20% single-valve surgery, 8% OPCAB, 5% modified Bentall procedure, 5% thoracic aneurysm operation, 5% combined operations, 5% other, 2% CABG+infarct exclusion	1991–2006: non-VAC2006–2010: VAC	NR	In-hospital	NR
Baillot2010^15^	Retrospective cohort; 2002–2007*, Canada	149 (125 vs 24)	(B) IMA	VAC or non-VAC; no reasons are reported	NR	In-hospital	NR
De Feo2010^17^	Retrospective cohort; 2000–2009, Italy	75 (45 vs 30)	Cardiac surgery	VAC or non-VAC; no reasons are reported	KCI system	NA	NR
Petzina2010^27^	Retrospective cohort; 2004–2009, Germany	118 (69 vs 49)	84% CABG ± valve procedure	2004–2006: non-VAC2006–2009: VAC	NR	In-hospital	NR
Eyileten2009^21^	Retrospective cohort; 2000–2007, Turkey	65 (33 vs 32)	75% CABG, 9% MVR, 5% MVR+CABG, 5% AVR, 2% AVR+CABG, 2% Bentall procedure	2000–2004: non-VAC2005–2007: VAC	KCI system	In-hospital	NR
Fuchs2005^23^	Retrospective cohort; 1998–2003, Germany	68 (35 vs 33)	Bypass operations, heart valve replacements	1998–2000: non-VAC2000–2003: VAC	KCI system	Undetermined	NR
Immer2005^24^	Retrospective cohort; 1998–2003, Switzerland	55 (38 vs 17)	76% CABG, 58% unilateral mammarian artery, 15% bilateral mammarian artery, 1% reoperation	VAC or non-VAC;§ no reasons are reported	KCI system	Undetermined	NR
Segers2005^30^	Retrospective cohort; 1992–2003, Netherlands	63 (29 vs 34)	64% CABG, 14% valve surgery, 21% CABG+valve surgery, 2% other	VAC or non-VAC; no reasons are reported	KCI system	30-day	NR
Sjögren2005^11^	Retrospective cohort; 1994–2003, Sweden	101 (61 vs 40)	72% CABG, 28% other procedures	1994–1998: non-VAC1999–2003: VAC	NR	90-day	County of Skåne Medical Science Fund, University Hospital of Lund Donation Funds
Domkowski2003^20^	Retrospective cohort; 1997–2002, United Kingdom	102 (96 vs 6)	Cardiac surgery	VAC or non-VAC; no reasons are reported	NR	In-hospital	NR
Doss2002^8^	Retrospective cohort; 1998–2000, Germany	42 (20 vs 22)	69% CABG, 14% CABG+AVR, 5% CABG+carotid endarterectomy, 5% CABG +concomitant left ventricular aneurysm resection, 5% isolated AVR, 2% CABG+ concomitant AVR+mitral valve repair	1998–1999: non-VAC1999–2000: both non-VAC and VAC, according to the surgeon’s discretion	KCI system	In-hospital	NR
Berg2000^16^	Retrospective cohort; 1989–1997, Netherlands	60 (31 vs 29)	35% CABG with venous graft, 58% CABG with IMA, 5% valve replacement, 2% CABG+valve replacement	VAC or non-VAC; according to the surgeon’s discretion	NR	In-hospital	NR

* For patients treated for sternal wound infections between 1997 and 2001, no mortality data was available and therefore, they were not included in the analysis.

§ In this study, in a few patients, despite the availability of the VAC, a non-VAC therapy has been chosen due to the poor quality of the sternum (fractured, white aspect, fragile) or on the clinical situation with incontrollable infection and hemodynamic instability.

### Mortality

Twenty studies (2233 patients) provided data on mortality.[8], [11], [14], [15], [16], [18], [19], [20], [21], [22], [23], [24], [25], [26], [27], [28], [30], [31], [32], [33] ^refs^ Nineteen studies reported on deep sternal wound infections (15 on mediastinitis,[11], [14], [15], [16], [18], [19], [20], [21], [23], [25], [26], [27], [28], [30], [32], [33] 2 on osteomyelitis[8], [25] and 2 did not specify the type of infections[24], [31]). One study included both deep (69%) and superficial (31%) sternal wound infections.[22] In 12 studies, VAC treated patients were compared with a historical control group not receiving VAC therapy,[11], [18], [19], [21], [22], [23], [25], [26], [27], [28], [31], [32] while in 2 other studies,[8], [16] the selection of therapy (VAC or non-VAC) was at the surgeon’s discretion, as both types of therapy were simultaneously available. Six studies did not provide the criteria for the selection of therapy in the enrolled patients.[14], [15], [20], [24], [30], [33] Twelve studies provided data for in-hospital mortality,[8], [14], [15], [16], [18], [20], [21], [26], [27], [31], [32], [33] 2 on 30-day[28], [30] and 2 studies on 90-day mortality,[11], [19] while 4 studies did not determine when mortality was assessed.[22], [23], [24], [25].

**Figure 2:**
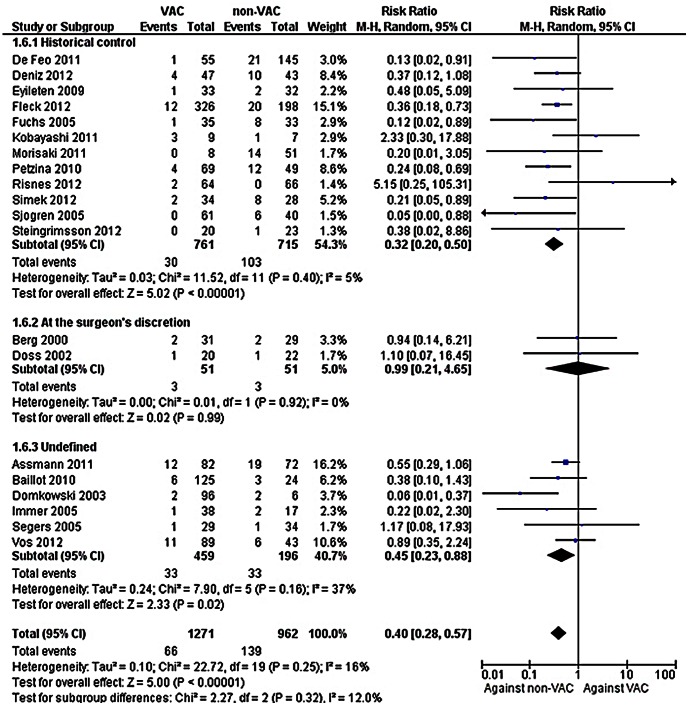
Forest plot depicting the risk ratios (RR) of mortality of patients according to the selection of the type of therapy. (*Vertical line = "no difference" point between the two regimens. Squares = risk ratios; Diamonds = pooled risk ratios for all studies. Horizontal lines = 95% CI*).

Pooling of all 20 studies that provided data on mortality showed that patients treated with VAC had significantly lower mortality compared to those treated with a non-VAC therapy, [Figure 2, 2233 patients, RR = 0.40, (95% CI: 0.28, 0.57)]. Heterogeneity was not observed in this analysis (*I* ^2^ = 16%). In addition, mortality was lower among patients receiving VAC therapy after the exclusion of studies that showed significantly lower mortality [1058 patients, RR 0.60, (95% CI: 0.41–0.89)]. Lower mortality was found among patients treated with VAC among studies using a historical non-VAC control group [1476 patients, RR = 0.32 (95% CI: 0.20, 0.50)]. Heterogeneity was not observed in this analysis (*I* ^2^ = 5%). Similarly, lower mortality was observed for VAC therapy in the studies that did not provide the criteria for the selection of therapy [655 patients, RR = 0.45 (95% CI: 0.23, 0.88)]. No difference was found between the compared groups when the selection of the type of therapy was at the surgeon’s discretion [102 patients, RR = 0.99, (95% CI: 0.21, 4.65)].

**Figure 3:**
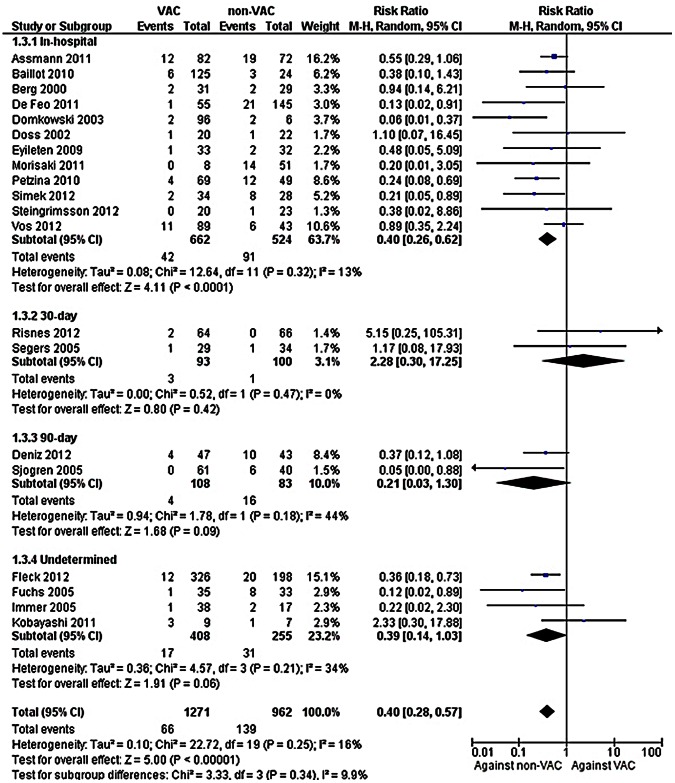
Forest plot depicting the risk ratios (RR) of mortality of patients according to the time of mortality assessment. (*Vertical line = "no difference" point between the two regimens. Squares = risk ratios; Diamonds = pooled risk ratios for all studies. Horizontal lines = 95% CI*).

In-hospital mortality was lower among patients treated with a VAC compared to those treated with a non-VAC therapy, [Figure 3, 1186 patients, RR = 0.40, (95% CI: 0.26, 0.62)]. Heterogeneity was not observed in this analysis (*I* ^2^ = 13%). On the other hand, no difference in 30-day [193 patients, RR = 2.28 (95% CI: 0.30, 17.25)] and 90-day mortality [191 patients, RR = 0.21 (95% CI: 0.03, 1.30)]; a small number of patients were included in these 2 analyses. Finally, in the studies that did not provide the time of mortality assessment a trend towards lower mortality was observed [663 patients, RR = 0.39 (95% CI: 0.14, 1.03)].

**Figure 4:**
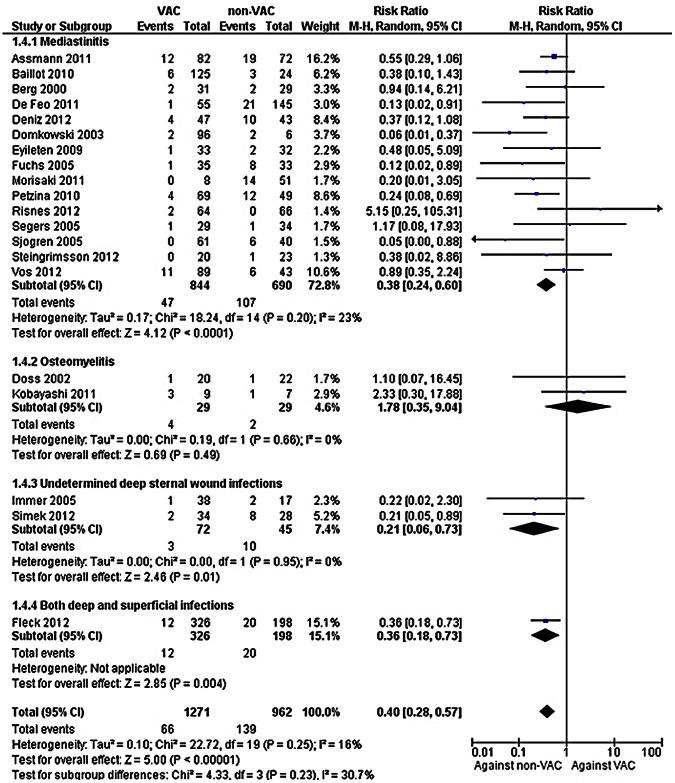
Forest plot depicting the risk ratios (RR) of mortality of patients according to the type of infection studied. (*Vertical line = "no difference" point between the two regimens. Squares = risk ratios; Diamonds = pooled risk ratios for all studies. Horizontal lines = 95% CI*).

Patients with mediastinitis and/or undetermined deep sternal wound infections treated with VAC therapy had lower mortality compared to the respective patients treated with a non-VAC therapy, [Figure 4, 1534 patients, RR = 0.38 (95% CI: 0.24, 0.60)] and [117 patients, RR = 0.21 (95% CI: 0.06, 0.73)] respectively. Heterogeneity was not observed in the abovementioned analyses (*I* ^2^ = 23% and *I* ^2^ = 0%, respectively). No difference in mortality was found between the compared groups among patients with osteomyelitis [58 patients, RR = 1.78 (95% CI: 0.35, 9.04)]. Last, 1 study including both deep and superficial sternal wound infections showed lower mortality among patients treated with VAC therapy than those treated with a non-VAC therapy [524 patients, RR = 0.36 (95% CI: 0.18, 0.73)].[22].

Only 4 of the 20 studies provided data regarding funding; 1 was industry-funded,[22] 2 were funded by universities[11], [32] while 1 study was not funded.[18] Therefore, potential bias arising out of funding could not be adequately investigated.

### Recurrences, Complications and Length of Stay

Ten studies provided data on recurrence of DSWIs (1197 patients).[11], [17], [21], [22], [25], [27], [28], [30], [31], [32] Pooling of these studies showed that recurrence was less common among patients treated with VAC compared to those treated with a non-VAC therapy, [RR = 0.34 (95% CI: 0.19, 0.59)]. Moderate heterogeneity was detected in this analysis (*I* ^2^ = 48%). Data on complications was available within 4 studies.[11], [14], [21], [31] Different types of complications were reported in the individual studies including remote infections, sepsis, cardiovascular/neurological/gastrointestinal complications, renal failure, bleeding, multiple organ failure, fistula, empyema, dehiscence, skin graft requirement, skin necrosis, seroma, discharging sinus, partial flap loss, new atrial fibrillation. However, only one study presented the total number of complications patients in each treatment arm,[14] while the remaining three studies presented the individual complications in each arm.[11], [21], [31] Therefore, the data could not be further analyzed.

Finally, ten studies provided data on LOS (983 patients).[8], [11], [14], [16], [19], [21], [27], [29], [31], [33] Pooling of the outcomes of these studies showed that there was no statistically significant difference in LOS between patients treated with VAC and those treated with a non-VAC therapy, [RR = −2.25 (95% CI: −7.52, 3.02)]. Considerable heterogeneity was detected in this analysis (*I* ^2^ = 82%) and individual studies showed that VAC was associated with both significantly lower and higher duration of hospitalization.

## Discussion

The currently available data from retrospective cohort studies suggest that the use of VAC therapy was associated with lower mortality than non-VAC therapy for the treatment of patients with DSWIs after cardiovascular surgery. This finding was consistently present regardless the study design, the inclusion of studies with positive findings, the criteria for establishment of the compared groups, the time of mortality assessment or the type of infections under study, provided that adequate data was available. In addition, VAC therapy was associated with fewer recurrences of infections. On the contrary, this meta-analysis did not show any difference in the duration of hospitalization.

The main limitation of the current meta-analysis is the retrospective nature of the available data. No randomized controlled trial has been published yet and one protocol had been registered –to our knowledge? regarding the effectiveness and safety of VAC therapy for the treatment of patients with DSWIs in which mortality is the primary end-point.[34] In addition, only one of the included studies performed a multivariate analysis to identify independent predictors for survival; VAC therapy was not introduced into this model and methicillin-resistant *Staphylococcus aureus* was the sole independent predictor for mortality.[26] Therefore, only unadjusted data were available for comparisons. A variety of techniques were used for the management of DSWIs in the control groups of both the individual studies and between studies; in addition, VAC was not the sole intervention applied in the VAC group of patients in all studies. As this clinical heterogeneity was expected, a random effect model was selected for all comparisons prior to the implementation of the meta-analysis. On the other hand, statistical heterogeneity was not observed in any of the performed analyses and all subgroup analyses consistently confirmed the results of the primary analysis, thus strengthening the validity of the results of the meta-analysis. Data regarding the offending bacteria and corresponding antibiotic treatment was not available. Finally, outcomes regarding the way of using VAC (i.e. pressure or duration) were not available within the included studies.

A recent international consensus conference suggested certain non-surgical interventions that are documented as decreasing mortality after a cardiac surgery and need further study;[35] administration of insulin, levosimendan, volatile anesthetics, statins, chronic beta-blockade, early aspirin therapy, the use of preoperative intra-aortic balloon counterpulsation are encountered among them. VAC therapy could be also included in this list if the lower mortality finding is confirmed in randomized studies. VAC therapy is not approved officially for the treatment of DSWIs. However, the positive findings of early studies showing lower mortality (although not uniformly) or decreased duration of hospitalization,[8], [11], [12], [16] in addition to better outcomes in favor of VAC therapy from RCTs in other patient populations, prompted the experts in the field to recommend the wider use of VAC for the treatment of patients with DSWIs.[1], [2] It should be noted that VAC is recommended “before primary closure, as preparation for secondary closure with vascularised tissue and as an adjunct to flap healing”.[1], [2].

Few studies provided data regarding recurrent DSWIs and even fewer for systemic or related to the surgical interventions complications. VAC therapy was associated with fewer recurrences than conventional treatment in the meta-analysis. Data regarding complications could not be further analyzed and adverse events following VAC and non-VAC therapy were not studied in this meta-analysis. In face of potentially lower mortality, the development of complications and adverse events seems negligible. However, surgeons should be aware of them in order to improve the quality of life of their patients. Complications of VAC treatment include bleeding (although sometimes its presence can be attributed to factors other than the VAC itself),[36] decrease of cardiac output when it is applied directly on the heart,[37] and adhesion formation and organ injury after the application of vacuum.[2] Advisory panels also warn against the use of VAC therapy when the patient has excessive or uncontrolled bleeding or uses anticoagulants that results in international normalized ratio over 2, untreated or undebrided osteomyelitis, and chest or pulmonary malignancy.[2].

Infections are among the major complications that prolong hospitalization. Controversial results were reported regarding this outcome in the studies included in the meta-analysis; six studies reported that LOS was significantly lower in patients receiving VAC therapy, while two reported that LOS was significantly prolonged. The meta-analysis including 10 studies showed no difference in the LOS when VAC was used, but considerable statistical heterogeneity was found. Therefore, it is difficult to draw conclusions regarding LOS. A recently published meta-analysis concluded that LOS was shortened with the use of VAC.[13] This meta-analysis included data from 6 studies; 3 of them were also included in the present meta-analysis. One did not provide the mean and standard deviation,[7] one provided the mean but not the standard deviation,[30] and one provided separate data for two groups of patients receiving VAC;[24] since we did not contact with the corresponding authors to request additional data, these data could not be included in the meta-analysis. Since hospital cost is associated mainly with LOS, these findings question the cost-effectiveness of VAC therapy in this patient population. Different findings in favor or against VAC regarding cost-effectiveness have been published.[38], [39], [40], [41].

In conclusion, the currently available data suggest a lower mortality and support the use of VAC therapy for the treatment of patients with DSWIs following cardiothoracic surgery. The retrospective design of the studies included in the meta-analysis and the lack of adjusted data highlighting VAC as an independent predictor of survival suggest that a well designed RCT is warranted to study the effects of VAC therapy, alone or in combination with other techniques, on mortality of patients with DSWIs. The effect of VAC on LOS and the related cost should be further investigated in this patient population.

## References

[1] Bovill E, Banwell PE, Teot L, Eriksson E, Song C, et al (2008) Topical negative pressure wound therapy: a review of its role and guidelines for its use in the management of acute wounds. Int Wound J 5: 511–529. 18808432 10.1111/j.1742-481X.2008.00437.x PMC7951631

[2] Fleck T, Gustafsson R, Harding K, Ingemansson R, Lirtzman MD, et al (2006) The management of deep sternal wound infections using vacuum assisted closure (V.A.C.) therapy. Int Wound J 3: 273–280. 17199763 10.1111/j.1742-481X.2006.00273.x PMC7951489

[3] Toumpoulis IK, Anagnostopoulos CE, Derose JJ Jr, Swistel DG (2005) The impact of deep sternal wound infection on long-term survival after coronary artery bypass grafting. Chest 127: 464–471. 15705983 10.1378/chest.127.2.464

[4] Armstrong DG, Lavery LA (2005) Negative pressure wound therapy after partial diabetic foot amputation: a multicentre, randomised controlled trial. Lancet 366: 1704–1710. 16291063 10.1016/S0140-6736(05)67695-7

[5] Joseph E, Hamori CA, S B, Roaf E, Swann NF, et al (2000) A prospective randomized trial of vacuum-assisted closure versus standard therapy of chronic non-healing wounds. Wounds 12: 60–67.

[6] Vuerstaek JD, Vainas T, Wuite J, Nelemans P, Neumann MH, et al.. (2006) State-of-the-art treatment of chronic leg ulcers: A randomized controlled trial comparing vacuum-assisted closure (V.A.C.) with modern wound dressings. J Vasc Surg 44: 1029–1037; discussion 1038. 10.1016/j.jvs.2006.07.030 17000077

[7] Catarino PA, Chamberlain MH, Wright NC, Black E, Campbell K, et al (2000) High-pressure suction drainage via a polyurethane foam in the management of poststernotomy mediastinitis. Ann Thorac Surg 70: 1891–1895. 11156090 10.1016/s0003-4975(00)02173-1

[8] Doss M, Martens S, Wood JP, Wolff JD, Baier C, et al (2002) Vacuum-assisted suction drainage versus conventional treatment in the management of poststernotomy osteomyelitis. Eur J Cardiothorac Surg 22: 934–938. 12467816 10.1016/s1010-7940(02)00594-8

[9] Gabriel A, Shores J, Heinrich C, Baqai W, Kalina S, et al (2008) Negative pressure wound therapy with instillation: a pilot study describing a new method for treating infected wounds. Int Wound J 5: 399–413. 18593390 10.1111/j.1742-481X.2007.00423.x PMC7951189

[10] Flack S, Apelqvist J, Keith M, Trueman P, Williams D (2008) An economic evaluation of VAC therapy compared with wound dressings in the treatment of diabetic foot ulcers. J Wound Care 17: 71–78. 18389832 10.12968/jowc.2008.17.2.28181

[11] Sjogren J, Gustafsson R, Nilsson J, Malmsjo M, Ingemansson R (2005) Clinical outcome after poststernotomy mediastinitis: vacuum-assisted closure versus conventional treatment. Ann Thorac Surg 79: 2049–2055. 15919308 10.1016/j.athoracsur.2004.12.048

[12] Sjogren J, Nilsson J, Gustafsson R, Malmsjo M, Ingemansson R (2005) The impact of vacuum-assisted closure on long-term survival after post-sternotomy mediastinitis. Ann Thorac Surg 80: 1270–1275. 16181853 10.1016/j.athoracsur.2005.04.010

[13] Damiani G, Pinnarelli L, Sommella L, Tocco MP, Marvulli M, et al (2011) Vacuum-assisted closure therapy for patients with infected sternal wounds: a meta-analysis of current evidence. J Plast Reconstr Aesthet Surg 64: 1119–1123. 21256819 10.1016/j.bjps.2010.11.022

[14] Assmann A, Boeken U, Feindt P, Schurr P, Akhyari P, et al (2011) Vacuum-assisted wound closure is superior to primary rewiring in patients with deep sternal wound infection. Thorac Cardiovasc Surg 59: 25–29. 21243568 10.1055/s-0030-1250598

[15] Baillot R, Cloutier D, Montalin L, Cote L, Lellouche F, et al (2010) Impact of deep sternal wound infection management with vacuum-assisted closure therapy followed by sternal osteosynthesis: a 15-year review of 23,499 sternotomies. Eur J Cardiothorac Surg 37: 880–887. 19880326 10.1016/j.ejcts.2009.09.023

[16] Berg HF, Brands WG, van Geldorp TR, Kluytmans-VandenBergh FQ, Kluytmans JA (2000) Comparison between closed drainage techniques for the treatment of postoperative mediastinitis. Ann Thorac Surg 70: 924–929. 11016335 10.1016/s0003-4975(00)01524-1

[17] De Feo M, Vicchio M, Nappi G, Cotrufo M (2010) Role of vacuum in methicillin-resistant deep sternal wound infection. Asian Cardiovasc Thorac Ann 18: 360–363. 20719787 10.1177/0218492310375854

[18] De Feo M, Vicchio M, Sante P, Cerasuolo F, Nappi G (2011) Evolution in the treatment of mediastinitis: single-center experience. Asian Cardiovasc Thorac Ann 19: 39–43. 21357316 10.1177/0218492310395789

[19] Deniz H, Gokaslan G, Arslanoglu Y, Ozcaliskan O, Guzel G, et al (2012) Treatment outcomes of postoperative mediastinitis in cardiac surgery; negative pressure wound therapy versus conventional treatment. J Cardiothorac Surg 7: 67. 22784512 10.1186/1749-8090-7-67 PMC3432617

[20] Domkowski PW, Smith ML, Gonyon DL Jr, Drye C, Wooten MK, et al (2003) Evaluation of vacuum-assisted closure in the treatment of poststernotomy mediastinitis. J Thorac Cardiovasc Surg 126: 386–390. 12928634 10.1016/s0022-5223(03)00352-0

[21] Eyileten Z, Akar AR, Eryilmaz S, Sirlak M, Yazicioglu L, et al (2009) Vacuum-assisted closure and bilateral pectoralis muscle flaps for different stages of mediastinitis after cardiac surgery. Surg Today 39: 947–954. 19882316 10.1007/s00595-008-3982-5

[22] Fleck T, Fleck M (2012) Negative pressure wound therapy for the treatment of sternal wound infections after cardiac surgery. Int Wound J. 10.1111/j.1742-481X.2012.01079.x PMC7950944 22943741

[23] Fuchs U, Zittermann A, Stuettgen B, Groening A, Minami K, et al (2005) Clinical outcome of patients with deep sternal wound infection managed by vacuum-assisted closure compared to conventional therapy with open packing: a retrospective analysis. Ann Thorac Surg 79: 526–531. 15680828 10.1016/j.athoracsur.2004.08.032

[24] Immer FF, Durrer M, Muhlemann KS, Erni D, Gahl B, et al (2005) Deep sternal wound infection after cardiac surgery: modality of treatment and outcome. Ann Thorac Surg 80: 957–961. 16122463 10.1016/j.athoracsur.2005.03.035

[25] Kobayashi T, Mikamo A, Kurazumi H, Suzuki R, Shirasawa B, et al (2011) Secondary omental and pectoralis major double flap reconstruction following aggressive sternectomy for deep sternal wound infections after cardiac surgery. J Cardiothorac Surg 6: 56. 21501461 10.1186/1749-8090-6-56 PMC3094378

[26] Morisaki A, Hosono M, Sasaki Y, Hirai H, Sakaguchi M, et al (2011) Evaluation of risk factors for hospital mortality and current treatment for poststernotomy mediastinitis. Gen Thorac Cardiovasc Surg 59: 261–267. 21484552 10.1007/s11748-010-0727-3

[27] Petzina R, Hoffmann J, Navasardyan A, Malmsjo M, Stamm C, et al (2010) Negative pressure wound therapy for post-sternotomy mediastinitis reduces mortality rate and sternal re-infection rate compared to conventional treatment. Eur J Cardiothorac Surg 38: 110–113. 20171898 10.1016/j.ejcts.2010.01.028

[28] Risnes I, Abdelnoor M, Veel T, Svennevig JL, Lundblad R, et al.. (2012) Mediastinitis after coronary artery bypass grafting: the effect of vacuum-assisted closure versus traditional closed drainage on survival and reinfection rate. Int Wound J. 10.1111/j.1742-481X.2012.01060.x PMC7950556 22925188

[29] Rodriguez Cetina Biefer H, Sundermann SH, Emmert MY, Rancic Z, Salzberg SP, et al.. (2012) Negative microbiological results are not mandatory in deep sternal wound infections before wound closure. Eur J Cardiothorac Surg 42: 306–310; discussion 310. 10.1093/ejcts/ezr326 22290924

[30] Segers P, de Jong AP, Kloek JJ, de Mol BA (2005) Poststernotomy mediastinitis: comparison of two treatment modalities. Interact Cardiovasc Thorac Surg 4: 555–560. 17670481 10.1510/icvts.2005.112714

[31] Simek M, Hajek R, Fluger I, Molitor M, Grulichova J, et al (2012) Superiority of topical negative pressure over closed irrigation therapy of deep sternal wound infection in cardiac surgery. J Cardiovasc Surg (Torino) 53: 113–120. 22231537

[32] Steingrimsson S, Gottfredsson M, Gudmundsdottir I, Sjogren J, Gudbjartsson T (2012) Negative-pressure wound therapy for deep sternal wound infections reduces the rate of surgical interventions for early re-infections. Interact Cardiovasc Thorac Surg 15: 406–410. 22691377 10.1093/icvts/ivs254 PMC3422957

[33] Vos RJ, Yilmaz A, Sonker U, Kelder JC, Kloppenburg GT (2012) Primary closure using Redon drains vs vacuum-assisted closure in post-sternotomy mediastinitis. Eur J Cardiothorac Surg. 10.1093/ejcts/ezs404 22885227

[34] Sood S, Gupta R (2012) Antibiotic resistance pattern of community acquired uropathogens at a tertiary care hospital in jaipur, rajasthan. Indian J Community Med 37: 39–44. 22529539 10.4103/0970-0218.94023 PMC3326806

[35] Landoni G, Augoustides JG, Guarracino F, Santini F, Ponschab M, et al (2011) Mortality reduction in cardiac anesthesia and intensive care: results of the first International Consensus Conference. HSR Proc Intensive Care Cardiovasc Anesth 3: 9–19. 23439940 PMC3484607

[36] van Wingerden JJ, Segers P, Jekel L (2011) Major bleeding during negative pressure wound/V.A.C.(R)–therapy for postsurgical deep sternal wound infection–a critical appraisal. J Cardiothorac Surg 6: 121. 21955731 10.1186/1749-8090-6-121 PMC3191481

[37] Conquest AM, Garofalo JH, Maziarz DM, Mendelson KG, Su Sun Y, et al (2003) Hemodynamic effects of the vacuum-assisted closure device on open mediastinal wounds. J Surg Res 115: 209–213. 14697285 10.1016/s0022-4804(03)00331-7

[38] Braakenburg A, Obdeijn MC, Feitz R, van Rooij IA, van Griethuysen AJ, et al.. (2006) The clinical efficacy and cost effectiveness of the vacuum-assisted closure technique in the management of acute and chronic wounds: a randomized controlled trial. Plast Reconstr Surg 118: 390–397; discussion 398–400. 10.1097/01.prs.0000227675.63744.af 16874208

[39] Mokhtari A, Sjogren J, Nilsson J, Gustafsson R, Malmsjo M, et al (2008) The cost of vacuum-assisted closure therapy in treatment of deep sternal wound infection. Scand Cardiovasc J 42: 85–89. 18273735 10.1080/14017430701744469

[40] Othman D (2012) Negative pressure wound therapy literature review of efficacy, cost effectiveness, and impact on patients' quality of life in chronic wound management and its implementation in the United kingdom. Plast Surg Int 2012: 374398. 22701169 10.1155/2012/374398 PMC3369418

[41] Soares MO, Dumville JC, Ashby RL, Iglesias CP, Bojke L, et al.. (2012) Methods to Assess Cost-Effectiveness and Value of Further Research When Data Are Sparse: Negative-Pressure Wound Therapy for Severe Pressure Ulcers. Med Decis Making. 10.1177/0272989X12451058 22927694

